# Factor structure of the Self-Regulation Questionnaire among adult learners from Poland, Serbia, Slovakia, and the Czech Republic

**DOI:** 10.1186/s41155-022-00241-z

**Published:** 2022-12-30

**Authors:** Jitka Vaculíková, Ilona Kočvarová, Jan Kalenda, Zuzana Neupauer, Marija Cvijetić Vukčević, Anna Włoch

**Affiliations:** 1 Research Centre of FHS, Tomas Bata University in Zlín, Zlín, Czech Republic; 2 Matej Bel University, Faculty of Education, Banská Bystrica, Slovakia; 3 University of Novi Sad, Faculty of Education, Sombor, Serbia; 4 Pedagogical University of Cracow, Cracow, Poland

**Keywords:** Self-regulation, Adult learners, Exploratory factor analysis (EFA), Confirmatory factor analysis (CFA), Measurement invariance

## Abstract

**Background:**

One of the most significant human qualities is the ability to develop, implement, and flexibly maintain planned behaviour in order to achieve one’s goals. Self-regulation has become a relatively well-researched area in the field of psychology and pedagogy. However, empirically valid and reliable instrument is still missing across European context. The primary goal of this research was to analyze the psychometric properties of the Czech version of the Self-Regulation Questionnaire (SRQ-CZ) among adult learners from Poland, Serbia, Slovakia, and the Czech Republic.

**Objective:**

The aim of the present study was to examine the factor structure and psychometric properties of the SRQ-CZ validated in the Czech educational context in a multi-cultural sample.

**Methods:**

A total of 1711 adult learners from European countries including Poland, Serbia, Slovakia, and the Czech Republic completed the SRQ-CZ. The first step to reviewing the validity of the SRQ-CZ included testing face validity. Furthermore, exploratory factor analysis (EFA) was performed on half the sample and confirmatory factor analysis (CFA) on the other half. Measurement invariance was conducted across gender, age, and country followed by the evaluation of the reliability of the final instrument.

**Results:**

EFA showed that a three-factor structure best fit the data. The originally proposed Impulse Control and Self-Direction are merged into one distinct factor, while Decision Making and Goal Orientation comprise the other two. Goodness-of-fit statistics yielded from CFA showed a good fit for the model, introducing a reliable and measurement invariant instrument.

**Conclusion:**

The present study used a diverse multi-cultural sample to explore the factorial structure and psychometric properties of the SRQ-CZ. A three-factor model was obtained as the result of the exploratory and confirmatory factor analyses. Further analysis aiming at measurement invariance, comparing the sample according to gender, age, and country, led to satisfactory results. The only exception was a lack of model fit in the case of Serbia. Although further psychometric evaluation of the SRQ-CZ is still needed, the presented results constitute significant findings, confirming instrument validity and utility as a measure of generalized self-regulation capacity across adult learners in European educational context.

**Supplementary Information:**

The online version contains supplementary material available at 10.1186/s41155-022-00241-z.

## Background

Although self-regulation is considered a crucial precondition of any learning, the COVID-19 world increased the requirements on adults’ ability to self-direct and self-regulate learning. Learners in both formal and nonformal education more frequently have to gain their knowledge and skills through digital learning environments, where they receive less direction and formative assessment from teachers and educators (Di Petro & Karpiński, 2021; Stanistreet et al., 2021; Waller et al., 2020). Therefore, they tend to be more dependent on their own ability to develop, implement, and flexibly maintain planned behaviour in order to achieve one’s goals (Brown et al., 1999).

Moreover, an unpredictable situation in the labour market, accompanied by the speed of social and technological changes, leads to the obsolescence of adults’ current skills and demand for new ones (ILO, 2021; OECD, 2019). Consequently, the need for lifelong learning and the ability to self-regulate learning activities and related behaviour has increased immensely (UNESCO, 2019, 2021; WEF, 2020).

These trends are not typical only for the Western world but also in Eastern Europe, which faces many new challenges in lifelong learning. These include the ageing of the population, the rising number of migrants that need to learn a new language and job-related skills, as well as a transformation of the industrial sector (Kalenda et al., 2022). However, most European countries lack a valid and reliable instrument for assessing the self-regulation of adult learners. Researchers and professionals cannot evaluate this crucial precondition of successful learning without such an instrument. For this reason, the primary purpose of this study is to culturally adapt a valid and reliable research instrument for measuring the self-regulation of adults in four European countries: Poland, Serbia, Slovakia, and the Czech Republic.

A number of theories explaining self-regulation processes have been developed to theoretically underline and empirically capture self-regulation activity across all continents and diverse fields of study (Boekaerts et al., 2005; Baumeister & Heatherton, 2009; Senécal & Vallerand, 1995; Veenstra et al., 2010). The conceptualisation of self-regulation has evolved from a rigid stimulus-response understanding of one’s capacity to behave independently but following an original external command (Diaz & Fruhauf, 1991) to a more complex conceptualisation including personality and social determinants (Brown et al., 1999). In the following paragraphs, we briefly elaborate the most important ones that are crucial for the theoretical background of the selected research instrument.

The first of these is a social cognitive perspective on self-regulation (Bandura, 1986, 1991) that highlights the interaction of personal, behavioural, and environmental processes. In this framework, self-regulation includes not only behavioural skills in managing environmental contingencies, but also contains a sense of personal agency to enact these skills in relevant contexts. This triadic model assumes that people self-regulate their behaviour through the use of their inner thoughts, feelings, and actions that are planned, monitored, and cyclically adapted according to acquired feedback concerning the effectiveness of strategies in meeting their reasonable goals. A person’s perception of self-efficacy plays a major role in motivating them to self-regulate their behaviour.

The second one is Carver and Scheier’s (1982; 1998) cybernetic self-regulation model of behaviour as a feedback loop. This conception focusses on a feedback cycle that is characterized by four phases summarized as TOTE (‘Test-Operate-Test-Exit’). In this cycle, a goal is first planned. Subsequently, a ‘Test’ is performed to identify whether the goal has been achieved. If not, ‘Operations’ are performed to achieve the goal. Finally, the Test is performed again. If there are no discrepancies between current and desired states, the individual enters the ‘Exit’ phase. Otherwise, the process repeats in a loop.

Synthesizing ideas from the previous two theoretical streams and elaborated on in Kanfer (1970) three-phase theory, Miller and Brown (1991) developed their own conception, including self-monitoring, self-evaluation, and self-reinforcement and extended the number of self-regulation processes to seven. Based on this theoretical framework, the authors developed the 63-item Self-Regulation Questionnaire (SRQ) to initially assess these self-regulatory processes (Brown et al., 1999). In addition to this research instrument, two others were developed in the same direction. Carey et al. (2004) provided a single-dimension solution that was adapted into the 31-item Short Self-Regulation Questionnaire (SSRQ), while Neal and Carey (2005a, b) proposed a two-factor solution as the measure of someone’s capacity for self-regulation.

Since the original SRQ (Brown et al., 1999) has been widely used and tested for psychometric properties in a variety of life domains, we believe that it is a useful research instrument for investigating the self-regulation abilities of adult learners. However, previous studies have established several factorial solutions. Out of three American studies (Bandura et al., 2003; Brown et al., 1999; Carey et al., 2004; Neal and Carey, 2005a, b), none met CFA goodness-of-fit indices for the tested seven-factor model. Similarly, in South African research (Potgieter & Botha, 2009), Brown et al. (1999) proposed model required modifications in order to meet analysis requirements. As a result, they formulated a six-factor solution with 24 items. Likewise, Pichardo et al. (2014; 2018) and Gavora et al. (2015) suggested a more parsimonious model with four factors. In summary, the SRQ lacks stable factorial structure and sufficient data fit, as predominantly measured on students’ samples.

For this reason, we remedy this gap with an empirical evaluation of the SRQ’s reliability and construct validity on a culturally diverse sample of adult learners while following applications and guidelines of cross-cultural psychology (Berry et al., 2013; McLean, 2022). Moreover, considering the culture-sensitive nature of self-regulation (Jaramillo et al., 2017; LeCuyer & Zhang, 2015), measurement invariance was conducted across each country of origin. Further, new insights are presented with respect to gender differences in self-regulation as widely reported from both the psychological (van Tetering et al., 2020; Velayutham et al., 2012) and neurological (Hosseini-Kamkar & Morton, 2014) perspectives. The study also investigates the finding that age represents an important factor in determining individual differences in self-regulation (Raffaelli et al., 2005). More specifically, we investigated whether the SRQ’s model structure for equivalency across groups of gender, age, and country by a series of measurement invariance tests.

The aim of this study is to describe the validation of the Czech version of the Self-Regulation Questionnaire (SRQ-CZ; Gavora et al., 2015) using cross-cultural data from adult learners with similar cultural backgrounds in four European countries: Poland, Serbia, Slovakia, and the Czech Republic. Thus, the main objective is to examine and ascertain whether the SRQ-CZ shows the same representation of self-regulation as previously reported by researchers (Gavora et al., 2015). On this basis, we present an evaluation of the face validity and construct validity of the instrument. To investigate the factorial structure of the instrument, EFA and CFA were used, followed by an evaluation of measurement invariance across gender, age, and country and then an evaluation of the reliability of the final instrument. The main contribution of this study is to broaden our understanding of self-regulation of behaviour among adult learners from an understudied European perspective.

## Method

### Participants

The research sample consisted of 1,711 adult learners from four European countries: Poland (*n* = 276), Serbia (*n* = 410), Slovakia (*n* = 511), and the Czech Republic (*n* = 514). Data collection was based on a convenient sample of learners enrolled in formal education, aged between 18 and 64 years. The questionnaire was administrated by a national research team in the first quarter of 2022 using an internet surveying technique or by a specialized agency using the Computer Assisted Web Interviewing method (CAWI).

The data collection and data analysis in this study have followed ethical principles of research, respecting the ICC/ESOMAR International Code (ESOMAR, 2016). The principle of anonymity was applied to maintain the anonymity of the participants, and the researchers emphasized informed consent throughout the study. Participants were informed about the aims of the research and that the given information would be treated confidentially. Furthermore, grant-project reviewers evaluated the grant proposals with respect to their ethical implications, ensuring the safety and rights of participants.

The whole sample consisted of 355 (20.7%) males with an average age of 26.36 years (*SD* = 7.9) and 1356 (79.3%) females with an average age of 25.1 years (*SD* = 7), with an overall mean age of 25.4 years (ranging from 18 to 61 years; *SD* = 7.3). Data on the highest attained educational level showed that the majority had attained at least three years of higher education and therefore earned the Bachelor degree (i.e. International Standard Classification of Education ISCED 6). Detailed demographic information about the samples can be seen in Table 1.

**Table 1:** Demographic details of the samples

Variables		Poland (*n* = 276)	Serbia (*n* = 410)	Slovakia (*n* = 511)	Czech Republic (*n* = 514)	Total (*n* =1711)
		*n* (%)				
Gender	Male	23 (8.3)	105 (25.6)	98 (19.2)	129 (25.1)	355 (20.7)
	Female	253 (91.7)	305 (74.4)	413 (80.8)	385 (74.9)	1356 (79.3)
Age	18–20 years	94 (34.1)	65 (15.9)	177 (34.6)	48 (9.3)	384 (22.4)
	21+ years	182 (65.9)	345 (84.1)	334 (65.4)	466 (90.7)	1327 (77.6)
Education	ISCED 3-5	123 (44.6)	152 (37.1)	0.0 (0.0)	204 (39.7)	479 (28.0)
	ISCED 6	87 (31.5)	134 (32.7)	350 (68.5)	236 (45.9)	807 (47.2)
	ISCED 7	62 (22.5)	95 (23.2)	120 (23.5)	73 (14.2)	350 (20.5)
	ISCED 8	4 (1.4)	29 (7.1)	41 (8)	1 (.20)	75 (4.4)

### Measure

The original SRQ (Brown et al., 1999) is a 63-item, self-reporting instrument designed to assess the ability for behavioural self-regulation in seven phases. These phases reflect the ability of individuals to receive relevant information, evaluate and compare it to norms, trigger change, search for options, formulate a plan, implement the plan, and assess the plan’s effectiveness. However, we decided to use the Czech version of the SRQ (SRQ-CZ; Gavora et al., 2015), based on cultural appropriateness for the target population of European learners. Factor analysis of the SRQ-CZ disconfirmed the original seven-phase theory and instead yielded a model with four factors: Impulse Control (8 items), Goal Orientation (5 items), Self-Direction (7 items), and Decision Making (7 items). In this study, the Czech version of SRQ included 27 items with response options on five-point Likert scale ranging from “strongly disagree” to “strongly agree” along equal intervals.

Participants of this study were informed that there are no right or wrong answers and they should respond to the items quickly without thinking too long. The results are calculated as the arithmetic mean to express the total raw score and the scores of individual dimensions. Despite the fact that the tool has already been validated, we decided to first apply exploratory and only then confirmatory factor analysis. The reason for this procedure is that the tool exists in several permutations with different numbers of factors and items, so its current form is not internationally stable.

Four necessary steps were involved in the instrument’s preparation (AERA et al., 1999; Hambleton et al., 2005; ITC, 2017). First, SRQ-CZ items were translated by skilled translators to produce the language modifications of the instrument for Polish, Serbian, and Slovak. Before the translation of the instrument, an online seminar was organised, wherein the research partners discussed and agreed on the basic standards of the translation process. Second, backward and forward translations were applied to produce the primary language modifications. These modifications were analysed by experienced researchers for the congruence of items with cultural traditions and critically assessed within the national research team. Lastly, each language version was field tested on a sample of the target population to judge the face validity. While researchers had a deep understanding of the background of the instrument, target participants provided valuable insights that otherwise might be missed. Focus was placed on the content and logical cohesion of the instrument. Detailed overview of the items is presented in Additional file 1.

### Procedure and data analyses

All analytical procedures were applied, along with judgment criteria which took into account the theoretical framework as well as the practical usefulness of the instrument. Individual steps assessing face and construct validity were evaluated simultaneously based on statistical as well as judgmental criteria focused on individual items and factors as well as the questionnaire as a whole. In this context, we empirically verified whether the SRQ-CZ had an identical structure, as indicated by previous research (Gavora et al., 2015), or whether another factorial solution can be identified, as suggested by predominantly non-European studies (Brown et al., 1999; Carey et al., 2004; Neal and Carey, 2005a, b; Potgieter & Botha, 2009). Furthermore, we evaluated measurement invariance according to gender, age, and country, any of which may play the role of a secondary factor influencing self-regulation capacity.

First, the inspection of the means, standard deviations, skewness, and kurtosis of each item was accomplished through item analysis. The factor structure of the SRQ-CZ was further cross-validated (James et al., 2013) using a sample randomly divided into two separate groups. Based on the hypothesized consistency of the correlated factors, EFA using Principal Component Analysis was applied to the item correlation matrix. Following the recommendations of Tabachninick and Fidell (2014), oblique promax rotation was applied as a suitable compromise (Russell, 2002).

To retain satisfactory variables, the .40–.30–.20 rule was adopted (Howard, 2016). In addition to consideration of the interpretable factor structure, we inspected Cattell’s scree plot (Cattell, 1966) and performed parallel analysis (Horn, 1965), as well as Wayne Velicer’s minimum average partial (MAP) analysis (Velicer, 1976). To evaluate the internal consistency of the full and subscale scores obtained from the EFA, Cronbach’s *α*, McDonald’s *ω*, and Gutmann’s *λ6* were compared with subscales expected to be reliable if the internal reliability coefficient is at least ≥ .70 (DeVellis, 2016; Gidron, 2013), while also taking into account the number of scale items. Pearson correlation with Bonferroni correction was used to control for multiple testing. To interpret the sizes of correlation coefficients, we use effect-size labels according to Cohen’s (1988, p. 79–81) conventions of *r* = .10 (small); *r* = .30 (medium); and *r* = .50 (large) correlation.

Second, a series of CFAs of the factor structure of the SRQ-CZ obtained in the previous stage was performed on the other half of the sample. The final model was also verified on separate samples divided by the gender, age, and country. Third, a series of measurement invariance tests using multiple group CFA were carried out to determine whether the model structure was equivalent across groups of gender, age, and country. Configural, metric, and scalar levels of invariance were evaluated.

Based on the common recommendations to investigate the model’s goodness of fit (Hooper et al., 2008), a number of statistics were used: comparative fit index (CFI; Bentler, 1990), Tucker–Lewis index (TLI; Tucker & Lewis, 1973), and root mean square error of approximation (RMSEA; Steiger, 1990). In addition, we reported the Chi-Square (*x*^*2*^) statistic, degrees of freedom, and its *p* value.

The descriptive item analysis, EFA, and the rawpar.sps and map.sps scripts (O´Connor, 2000; Velicer, 1976) were undertaken in IBM SPSS 27.0. IBM SPSS AMOS 27.0 was used to perform CFA and MI. We also used JASP 0.16.2.0 to calculate Cronbach’s *α*, McDonald’s *ω*, and Gutmann’s *λ6*. Only responses with no missing values were included in the analysis.

## Results

Descriptive statistics of the SRQ-CZ can be seen in Additional file 1. The means of all 27 items ranged between 2.39 and 4.12 out of 5, with a mean score of 3.28. The values of the standard deviations (*SD*) of all items ranged between .89 to 1.28, with a mean of 1.08. Values of the skewness and kurtosis of all items did not exceed the value of ≥ 2 for skewness and ≥ 7 for kurtosis (Curran et al., 1996; Trochim & Donnelly, 2006), suggesting no serious violation of the data dispersion. In the next step, the research sample was randomly divided into two independent samples for the EFA and CFA, respectively. The 27 items of self-regulation were subjected to EFA with the aim of gathering information about the interrelationships among the set of variables.

### Exploring the factor structure of the SRQ-CZ

The Kaiser-Meyer-Olkin measure of sampling adequacy (KMO) was .89, indicating that the sample was adequate, and Bartlett’s test of sphericity reached significance (*x*^*2*^(351) = 6739.62, *p* < .001), supporting the factorability of the correlation matrix.

A scree plot of eigenvalues (not shown) was strongly in favour of the three-factor structure. This result was supported by the parallel analysis, indicating the presence of three components with eigenvalues exceeding the corresponding criterion values of a randomly generated data matrix. Furthermore, Wayne Velicer’s original MAP test (Velicer, 1976) presented a three-factor structure. The number of components was also three according to the revised test version (O’Connor, 2000). Comparing these findings and the theory-based initiative with the data-driven solution, a three-factor structure of the SRQ-CZ was selected.

A total of 22 items were shown to remain by the results of EFA, from which three scales were created, accounting for approximately 41% of the variance, with eigenvalues of 6.63, 2.88, and 1.56 respectively. The mean of the final items on a split sample used for EFA (*n* = 855) ranged between 2.42 and 4.05 (*SD* = .90 to 1.30). The item that best explained the variance measured in the participant’s self-regulation was GO19 (*I have rules that I stick by no matter what*), from the third factor. On the other hand, item GO10 (*I can stick to a plan that’s working well*) from the same factor had the lowest factor loading, communalities, and a lower average correlation than the rest of the items. Based on the theoretical framework of SRQ, item GO10 well represented the meaning of the factor. Moreover, we tested a model without this item; however, it did not reach better fit. Therefore, we decided to retain the item within the factor and support multiple-factor indicators (at least four to six per factor), performing analysis with largely determined factors (Fabrigar et al., 1999). The factor loadings of each item as well as reliability are presented in Table 2.

**Table 2:** Pattern matrix of the SRQ-CZ (*n* = 855)

Item description	F1	F2	F3	*h*^*2*^	*α-i*
IC7: I have trouble following through with things once I’ve made up my mind to do something.	**.790**	−.036	.067	.611	.831
IC3: I get easily distracted from my plans.	**.708**	.244	−.139	.497	.841
IC9: I can come up with lots of ways to change, but it's hard for me to decide which one to use.	**.698**	.159	−.017	.440	.842
IC27: I give up quickly.	**.685**	−.121	.046	.517	.838
SD4: I don’t notice the effects of my actions until it’s too late.	**.658**	.002	−.047	.456	.841
IC8: I don’t seem to learn from my mistakes.	**.634**	−.022	−.010	.417	.844
IC6: When it comes to deciding about a change, I feel overwhelmed by the choices.	**.610**	−.003	.110	.338	.848
SD5: It’s hard for me to see anything helpful about changing my ways.	**.603**	−.078	.115	.359	.847
SD15: I have a hard time setting goals for myself.	**.588**	−.101	−.051	.427	.844
SD2: I have trouble making up my mind about things.	**.571**	.147	.015	.284	.854
IC21: Often I don’t notice what I’m doing until someone calls it to my attention.	**.571**	.070	.058	.286	.838
DM23: I’m good at finding different ways to get what I want.	.017	**.742**	−.088	.490	.706
DM14: As soon as I see a problem or challenge, I start looking for possible solutions.	−.138	**.684**	−.049	.521	.704
DM20: I can usually find several different possibilities when I want to change.	.007	**.669**	.004	.447	.712
DM18: There is usually more than one way to accomplish something.	.096	**.650**	.017	.396	.724
DM16: When I’m trying to change something, I pay a lot of attention to how I’m doing.	.193	**.649**	−.041	.353	.737
DM17: As soon as I see things aren’t going right I want to do something about it.	−.147	**.583**	.038	.448	.716
DM22: Usually I see the need to change before others do.	.188	**.489**	.036	.221	.759
GO19: I have rules that I stick by no matter what.	.080	−.148	**.903**	.680	.631
GO13: I am set in my ways.	.048	−.033	**.841**	.658	.612
GO12: I have personal standards, and try to live up to them.	.085	.092	**.756**	.599	.652
GO10: I can stick to a plan that’s working well.	−.186	.109	**.390**	.303	.782
Factor label	SC	DM	GO	Together	
No. of items	11	7	4	22	
*M*	2.70	3.60	3.72	3.34	
*SD*	.78	.63	.77	.73	
Explained variance in %	24.56	10.68	5.76	41	
McDonald’s *ω*	.858	.858	.758	.436	
Cronbach’s *α*	.856	.856	.737	.676	
Gutmann’s *λ6*	.853	.853	.696	.758	

*Note:* The extraction method was principal component analysis; the rotation method was promax with Kaiser Normalization. Factor loadings in bold represent items’ loadings onto their primary factor. F1–3 = factor, *h*^*2*^ = communalities, α*-i* = Cronbach’s *α* if the item is deleted. *SC* Self-Control, *DM* Decision Making, *GO* Goal Orientation

The first subscale, titled Self-Control, consisted of eleven items assessing internal self-control skills, including items initially falling under the factors Impulse Control (7 items, e.g. *I have trouble following through with things once I’ve made up my mind to do something*) and Self-Direction (4 items, e.g. *I don’t notice the effects of my actions until it’s too late*). The second subscale represented a factor measuring personal Decision Making (7 items, e.g. *I’m good at finding different ways to get what I want*). The third subscale comprised four goal orientation-related items (e.g. *I have rules that I stick by no matter what*).

The three subscales showed a medium correlation with each other, reaching expected directions. The correlations of the three subscales with the overall construct ranged from medium to large, which suggests the possibility of connecting all the subscales into one overall construct, as well as supporting a three-factor structure (see Additional file 2). At this point, we decided to further evaluate the connection of subscales into three factors.

### Evaluating the factor structure of the SRQ-CZ

In order to evaluate the data-driven foundation of the SRQ-CZ, we verified the construct validity of individual factors as well as their connection with the default model (see Additional file 3) on the other randomly divided sample (*n* = 856). The results of the CFA model fit are displayed in Table 3.

**Table 3:** CFA goodness-of-fit statistics for the individual factors and default model (*n* = 856)

Model	No	*x*^2^	*df*	CFI	TLI	RMSEA
F1 (Self-Control)	11	140.035	44	.967	.959	.050
F2 (Decision Making)	7	44.512	14	.977	.965	.050
F3 (Goal Orientation)	4	9.294	2	.992	.975	.065
Default model	22	665.179	206	.920	.911	.051

*Note:* Self-Control includes the Impulse Control and Self-Direction items. No = number of items; *p* < .001

As can be seen in Table 3, all tested independent factors fulfilled the statistical as well as the judgment criteria for the tool construction. This means that the factors work independently well and can be applied separately. Moreover, the connection of the factors under the umbrella of one default model also proved to be functional: *x*^*2*^(206) = 665.179, CFI = .920, TLI = .911, RMSEA = .051.

### Measurement invariance of the SRQ-CZ across gender, age, and country

According to Cheung and Rensvold’s (2002) recommendation of the differences among the values of ΔCFI (with values lower than .01 considered a sign of invariance), the factor means of males and females can be compared up to the scalar level. Similarly, the difference between the age groups is measurement invariant up to the scalar level. Nevertheless, based on the results shown in Table 4, the instrument does not seem to be invariant across countries.

**Table 4:** Measurement invariance of the default model for predefined groups (*n* = 856)

Grouping variable	Level of invariance	*x*^2^	*df*	CFI	ΔCFI	TLI	RMSEA
Gender	Configural	900.514	412	.916		.905	.053
	Metric	916.414	431	.916	0	.910	.051
	Scalar	961.789	450	.912	-.004	.909	.052
Age	Configural	899.548	412	.916		.906	.053
	Metric	920.445	431	.916	0	.909	.052
	Scalar	938.434	450	.916	0	.913	.050
Country	Configural	1560.571	824	.881		.867	.065
	Metric	1681.58	881	.871	-.01	.865	.065
	Scalar	2215.514	938	.794	-.077	.797	.080

*Note: p* < .001

We additionally checked the CFA goodness-of-fit statistics for the default model independently across country samples (see Additional file 4). All the countries reached acceptable parameters except Serbia, for which the indices (CFI = .871, TLI = .856) remained below the recommended value of .90 (Bentler, 1990). For this reason, we further compared measurement invariance across countries without the sample from Serbia (see Additional file 5). The default model was invariant up to the configural model, which represents the testing of the factor structure. Invariant factor loadings (metric model) and the intercepts constrained to be equal (scalar model) showed poor fit.

### Reliability of the SRQ-CZ

In addition to the presented results of construct validity, the reliability coefficients were calculated. Besides Cronbach’s *α* reliability coefficient, which while commonly used is no longer sufficiently warranted as a sole index of reliability (Agbo, 2010), McDonald’s *ω* and Gutmann’s *λ6* were calculated. As can be seen in Additional file 6, the three factors of the default model are expected to be reliable in all monitored groups. In other words, all indices showed good reliability (≥ .70) for the three factors across samples with the exception of the internal consistency of the Goal Orientation factor (*λ6* = .663) in the case of Serbia sample. With regard to Serbia, however, this factor did reach satisfactory results in the case of Cronbach’s *α* = .705, and McDonald’s *ω* = .722.

## Discussion

The main objective of the present study was to describe the construct validity of the Czech version of the Self-Regulation Questionnaire (SRQ-CZ), aimed at measuring the self-regulation of behaviour in four phases. Based on findings from the sample of adult learners in Poland, Serbia, Slovakia, and the Czech Republic, this study represents a unique cross-cultural approach in understanding the personal ability to act according to an internal plan to achieve personal goals, without external support or reward.

In line with previous research (Gavora et al., 2015), we provide empirical support for the existence of Impulse Control and Self-Direction within the Self-Control factor, and Decision Making and Goal Orientation within separate factors, evaluated on a culturally diverse sample. The significance of this research is the finding that the instrument holds the same constructs of behaviour self-regulation as reported in the pioneering work of Gavora et al. (2015). The only exception is the model’s modification in the case of item purification (from 22 to 27 items) and factor reduction (from three to four factors). On this basis, Impulse Control and Self-Direction, measuring the inhibition of emotive response tendencies, created one common factor called Self-Control. Items included in this shared factor were similar in the nature of their meanings, as well as in their reversed polarity, which could significantly influence respondents’ pattern of responses. When comparing this factor to the remaining constructs (Decision Making and Goal Orientation), factor scores need to be re-coded to report on the negative function of self-regulation.

Overall, we have found that the final model of the instrument is robust in its application as a whole, as well as in the separate usage of all individual factors. Another important finding of our study is the suitability of the instrument for the specific groups of respondents divided by gender and age. In practise, it supports the statistical relevance of mean comparison of self-regulation of behaviour between males and females and groups divided by age.

The obtained model was further tested for measurement invariance according to country of origin. In the case of Serbia, the results showed that the final model of the instrument might be weak. Screening the data, distributional properties and demographic information about the Serbian sample did not vary from the specified population of interest, i.e. did not vary from other national samples used in this study. Thus the question remains as to how specific the Serbian perception of self-regulated behaviour is as compared to other Central and Eastern European countries, as well as how Serbian perceptions of self-regulation differ from that of the neighbouring countries of Poland, Slovakia, and the Czech Republic, all of which are similar culturally and demographically. Due to this discrepancy, application of the instrument without further investigation would be inappropriate at this stage of the research. We also tested measurement invariance on the rest of the countries in the analysis (without Serbia), showing that the same structure of the model holds for all groups (invariant on the configural level). Further modifications of the model would be needed to increase the international comparability of the instrument. However, it should be noted that the instrument was evaluated as internally consistent across all the created samples, including the acceptable values (Cronbach’s *α* and McDonald’s *ω*) of Serbia

Pichardo-Martínez et al. (2014) reported the implementation of a research strategy similar to this study. In their study, 63 items from the SRQ were administrated to a sample of 834 students randomly divided into two sets of exploratory and confirmatory samples. The results show evidence for the validity and internal consistency of the Short Self-Regulation Questionnaire (SSRQ) in the Spanish context, suggesting a four-factor model with 17 items. Likewise, data from 845 Spanish early and middle adolescents showed goodness of fit with the four-factor model and the same number of items (Pichardo et al., 2018). However, the socio-demographic and cultural diversity of the sample still needs research.

The present study has strengths, as well as some limitations, which should be addressed in future research. The relatively large sample size made it possible to randomly divide the data in half so that both EFA and CFA could be undertaken. The fact that the sample consisted of four national samples may be seen both as a strength and as a weakness. Due to the low response rate most likely justified by the onset of COVID-19 pandemic, it was not possible to conduct representative quota samples. Therefore, the presented results should not be assumed to be generalizable to the target population. On the other hand, this study represents one of the few attempts to research self-regulation from an international point of view and meet the recommendations for sample size when conducting CFA. It should be noted that our sample as a whole represents a highly educated population, based on their higher-than-average level of completed education. More than 70% of the participants reported having at least 3 years of university education (up to ISCED 6) and are now continuing to participate at a higher level.

This study included cultural and linguistic diversity that poses significant challenges to the process of standardization of measures (Hambleton & Zenisky, 2011; Sireci, 2011). Taking measurement equivalence across cultures and languages into full account, the biases of result interpretation were carefully evaluated using guidelines for adapting educational and psychological measures (Hambleton et al., 2005; ITC, 2017). Another question is related to the general character of the SRQ. It captures generic rather than specific tasks. We believe, however, that while filling in the questionnaire, respondents always relate their answers to a specific action in a life situation. For example, the results of a respondent who filled in the questionnaire related to self-regulation in sports may yield a different picture of self-regulation than that of a respondent who related their self-regulation to study activities. This questions the construct validity of the questionnaire.

As for future research, a chronology of the self-regulated behaviour, delivered from the seven-phase self-regulation theory (Miller & Brown, 1991), indicating whether a person systematically proceeds one step at a time and whether they use all activities in each phase, can be anticipated in a follow-up investigation. Based on the presented results, the SRQ-CZ can be further modified, extended, or used to supplement data from other surveys across diverse national or international samples. Moreover, it can be administrated by adult learning researchers, managers, lecturers, employers, consultants, or other professionals in formal educational settings for adult learners in European and non-European cultures (Aubrey et al., 1994; Carey et al., 2004; Neal and Carey, 2005a, b).

## Conclusions

The present study used a diverse multicultural sample to explore the factorial structure of the SRQ originally developed by Brown et al. (1999). This instrument assesses individuals’ ability for self-regulation in seven phases. Despite previous work on the instrument’s validation in the Czech context, we were unable to find a good model fit for a previously validated four-factor model (Gavora et al., 2015) using a multiple-methods design. The same factors appeared in our data but merged into a three-factor solution, with Impulse Control and Self-Direction in a separate factor called Self-Control, and Decision Making and Goal Orientation as the other two. Since previous studies have also failed to reach conclusive results on the optimal factor structure for the SRQ, further research is needed in order to disentangle the possible effects of gender, age, and nationality on self-regulation of behaviour. Until this evidence exists, it would be wise to conduct factor analysis in order to explore and evaluate whether the total score, and a three-factor structure, is applicable for their samples.

## Supplementary Information

**Additional file 1. **Overview of the SRQ-CZ items including descriptive statistics (*n* = 1,711).

**Additional file 2. **Correlation between SRQ-CZ subscales and the full scale (*n* = 855).

**Additional file 3. **Evaluated factor structures of a correlated three-factor model (*n* = 856).

**Additional file 4.** CFA goodness-of-fit statistics for the default model across countries.

**Additional file 5.** Measurement invariance of the default model for country.

**Additional file 6.** Reliability of the default model across samples.

## Data Availability

The datasets used and analysed during the current study are available from the corresponding author on reasonable request.

## Abbreviations

CFA: Confirmatory factor analysis
CFI: Comparative fit index
*df*: Degree of freedom
DM: Decision Making
EFA: Exploratory factor analysis
GO: Goal Orientation
*h*^*2*^: Communalities
ISCED: International Standard Classification of Education
KMO: Kaiser-Meyer-Olkin measure
M: Mean
MAP: Minimum average partial analysis
RMSEA: Root mean square error of approximation
SC: Self-Control
SD: Standard deviation
SRQ: Self-Regulation Questionnaire
SRQ-CZ: Czech Self-Regulation Questionnaire
TLI: Tucker–Lewis index
*x*^2^: Chi-square
*α-i*: Cronbach’s α if the item is deleted
