# Supplementary material for: Factor structure of the Self-Regulation Questionnaire among adult learners from Poland, Serbia, Slovakia, and the Czech Republic
Source: Psicol Reflex Crit. 2022 Dec 30;35:40. doi: 10.1186/s41155-022-00241-z (PMC9801149; doi:10.1186/s41155-022-00241-z)
Supplement: Supplementary file 1 — Additional file 1. Overview of the SRQ-CZ items including descriptive statistics (n = 1,711). [file 41155_2022_241_MOESM1_ESM.docx]

**Additional file 1**

Overview of the SRQ-CZ items including descriptive statistics (*n* = 1,711)

| Theoretical structure | Basic factor description | Item description | Descriptive statistics | | | |
| --- | --- | --- | --- | --- | --- | --- |
|  |  |  | *M* | *SD* | Skewness | Kurtosis |
| Impulse Control (IC) | Control of emotions or behaviors | IC1: I usually keep track of my progress toward my goals. | 4.02 | .890 | -.670 | -.026 |
|  |  | IC2: I have trouble making up my mind about things. | 2.48 | 1.275 | .445 | -.920 |
|  |  | IC3: I get easily distracted from my plans. | 2.58 | 1.203 | .353 | -.802 |
|  |  | IC5: It's hard for me to see anything helpful about changing my ways. | 2.41 | 1.126 | .450 | -.621 |
|  |  | IC6: When it comes to deciding about a change, I feel overwhelmed by the choices. | 3.43 | 1.216 | -.333 | -.872 |
|  |  | IC7: I have trouble following through with things once I've made up my mind to do something. | 2.71 | 1.233 | .248 | -.961 |
|  |  | IC9: I can come up with lots of ways to change, but it's hard for me to decide which one to use. | 2.87 | 1.218 | .075 | -.982 |
|  |  | IC17: I give up quickly. | 2.46 | 1.174 | .424 | -.717 |
| Goal Orientation (GO) | Completion of specific goals | GO10: I can stick to a plan that's working well. | 4.01 | .932 | -.857 | .484 |
|  |  | GO12: I have personal standards, and try to live up to them. | 3.95 | .970 | -.815 | .262 |
|  |  | GO13: I am set in my ways. | 3.63 | 1.045 | -.455 | -.432 |
|  |  | GO19: I have rules that I stick by no matter what. | 3.67 | 1.070 | -.550 | -.285 |
|  |  | GO26: I know how I want to be. | 4.12 | 1.023 | -1.112 | .657 |
| Self-Direction (SD) | Management of actions without external forces | SD4: I don't notice the effects of my actions until it's too late. | 2.70 | 1.147 | .171 | -.826 |
|  |  | SD8: I don't seem to learn from my mistakes. | 2.39 | 1.231 | .587 | -.645 |
|  |  | SD11: I usually only have to make a mistake one time in order to learn from it. | 3.15 | 1.185 | -.048 | -.881 |
|  |  | SD15: I have a hard time setting goals for myself. | 2.51 | 1.264 | .427 | -.905 |
|  |  | SD21: Often I don't notice what I'm doing until someone calls it to my attention. | 2.51 | 1.116 | .400 | -.598 |
|  |  | SD24: I usually think before I act. | 3.82 | 1.036 | -.721 | -.017 |
|  |  | SD25: I learn from my mistakes. | 3.83 | .949 | -.611 | -.034 |
| Decision Making (DM) | Selection of choices by identifying a decision,  and assessing alternative resolutions | DM14: As soon as I see a problem or challenge, I start looking for possible solutions. | 3.71 | 1.036 | -.547 | -.339 |
|  |  | DM16: When I'm trying to change something, I pay a lot of attention to how I'm doing. | 3.41 | 1.073 | -.384 | -.428 |
|  |  | DM17: As soon as I see things aren't going right I want to do something about it. | 3.79 | .937 | -.534 | -.075 |
|  |  | DM18: There is usually more than one way to accomplish something. | 4.06 | .888 | -.776 | .292 |
|  |  | DM20: I can usually find several different possibilities when I want to change | 3.68 | .946 | -.451 | -.205 |
|  |  | DM22: Usually I see the need to change before others do. | 3.21 | 1.049 | -.113 | -.481 |
|  |  | DM23: I'm good at finding different ways to get what I want. | 3.44 | 1.041 | -.268 | -.514 |

*Note:* Source of original items = SRQ (Brown et al., 1999). *M* = Mean; *SD* = Standard Deviation.
