# Supplementary material for: Factor structure of the Self-Regulation Questionnaire among adult learners from Poland, Serbia, Slovakia, and the Czech Republic
Source: Psicol Reflex Crit. 2022 Dec 30;35:40. doi: 10.1186/s41155-022-00241-z (PMC9801149; doi:10.1186/s41155-022-00241-z)
Supplement: Supplementary file 2 — Additional file 2. Correlation between SRQ-CZ subscales and the full scale (n = 855). [file 41155_2022_241_MOESM2_ESM.docx]

**Additional file 2**

Correlation between SRQ-CZ subscales and the full scale (*n* = 855)

| Suscales | Self-Control | Decision Making | Goal Orientation |
| --- | --- | --- | --- |
| Decision Making | -.264^**^ |  |  |
| Goal Orientation | -.304^**^ | .405^**^ |  |
| Together | .732^**^ | .387^**^ | .265^**^ |

*Note:* Correlation is significant at the .01 level (2-tailed).
