# Supplementary material for: Factor structure of the Self-Regulation Questionnaire among adult learners from Poland, Serbia, Slovakia, and the Czech Republic
Source: Psicol Reflex Crit. 2022 Dec 30;35:40. doi: 10.1186/s41155-022-00241-z (PMC9801149; doi:10.1186/s41155-022-00241-z)
Supplement: Supplementary file 3 — Additional file 3. Evaluated factor structures of a correlated three-factor model (n = 856). [file 41155_2022_241_MOESM3_ESM.docx]

**Additional file 3**

Evaluated factor structures of a correlated three-factor model (*n* = 856)


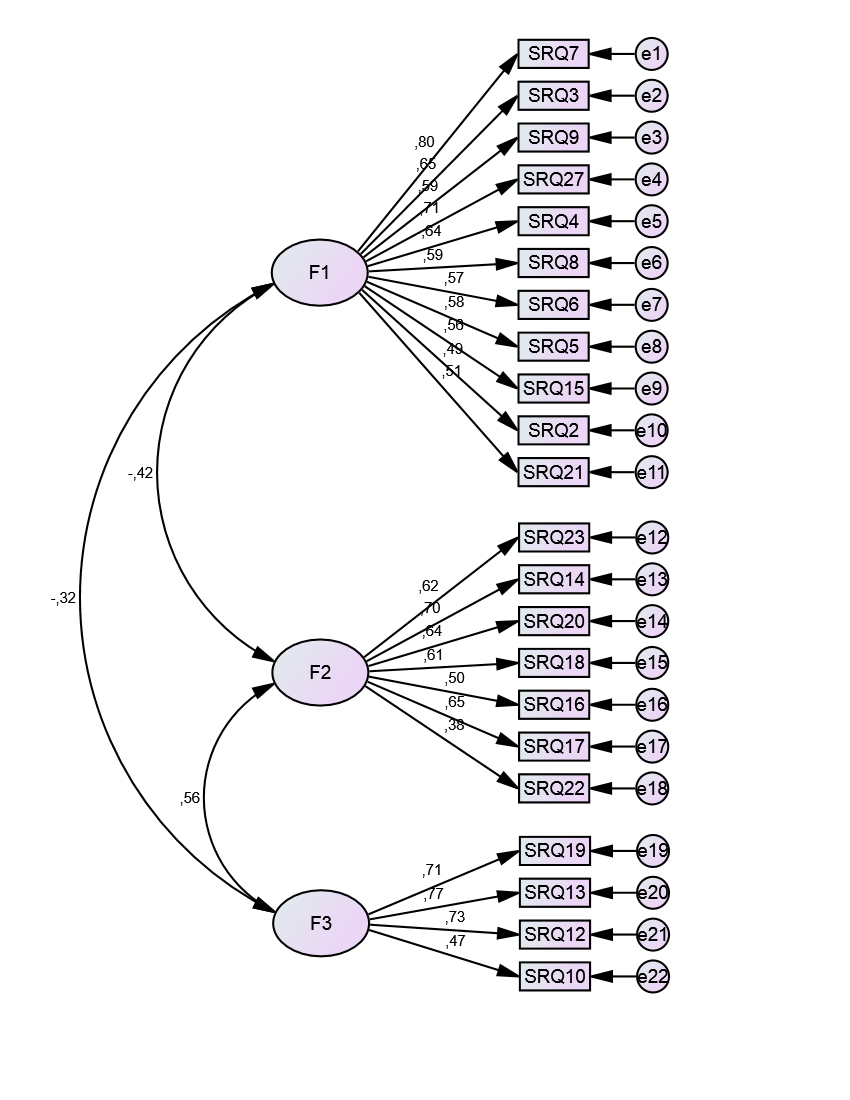


*Note.* Factor F1: Self-Control; F2: Decision Making; F3: Goal Orientation
