# Supplementary material for: Factor structure of the Self-Regulation Questionnaire among adult learners from Poland, Serbia, Slovakia, and the Czech Republic
Source: Psicol Reflex Crit. 2022 Dec 30;35:40. doi: 10.1186/s41155-022-00241-z (PMC9801149; doi:10.1186/s41155-022-00241-z)
Supplement: Supplementary file 4 — Additional file 4. CFA goodness-of-fit statistics for the default model across countries. [file 41155_2022_241_MOESM4_ESM.docx]

**Additional file 4**

CFA goodness-of-fit statistics for the default model across countries

| Country | N | *x^2^* | *df* | CFI | TLI | RMSEA |
| --- | --- | --- | --- | --- | --- | --- |
| Poland | 276 | 371.274 | 206 | .914 | .903 | .054 |
| Slovakia | 511 | 467.905 | 206 | .920 | .910 | .050 |
| Serbia | 410 | 552.103 | 206 | .871 | .856 | .064 |
| Czech Republic | 514 | 561.099 | 206 | .905 | .900 | .058 |

*Note:* *p* < .001.
