# Supplementary material for: Factor structure of the Self-Regulation Questionnaire among adult learners from Poland, Serbia, Slovakia, and the Czech Republic
Source: Psicol Reflex Crit. 2022 Dec 30;35:40. doi: 10.1186/s41155-022-00241-z (PMC9801149; doi:10.1186/s41155-022-00241-z)
Supplement: Supplementary file 5 — Additional file 5. Measurement invariance of the default model for country. [file 41155_2022_241_MOESM5_ESM.docx]

**Additional file 5**

Measurement invariance of the default model for country

*Note:* *Poland, Slovakia, and the Czech Republic; *p* < .001.

| Grouping variable | Level of invariance | *x^2^* | *df* | CFI | ΔCFI | TLI | RMSEA |
| --- | --- | --- | --- | --- | --- | --- | --- |
| Country* | Configural | 1082.111 | 738 | .902 |  | .890 | .059 |
|  | Metric | 1170.91 | 656 | .891 | -.011 | .885 | .060 |
|  | Scalar | 1402.792 | 694 | .850 | -.041 | .850 | .068 |
