# Supplementary material for: Factor structure of the Self-Regulation Questionnaire among adult learners from Poland, Serbia, Slovakia, and the Czech Republic
Source: Psicol Reflex Crit. 2022 Dec 30;35:40. doi: 10.1186/s41155-022-00241-z (PMC9801149; doi:10.1186/s41155-022-00241-z)
Supplement: Supplementary file 6 — Additional file 6. Reliability of the default model across samples. [file 41155_2022_241_MOESM6_ESM.docx]

**Additional file 6**

Reliability of the default model across samples

| Sample | Factor | Cronbach’s *α* | McDonald’s *ω* | Gutmann’s  *λ6* |
| --- | --- | --- | --- | --- |
| All groups  (*n* = 1.711) | F1: Self-Control | .863 | .865 | .859 |
|  | F2: Decision Making | .769 | .773 | .752 |
|  | F3: Goal Orientation | .763 | .779 | .720 |
| EFA sample  (*n* = 855) | F1: Self-Control | .856 | .858 | .853 |
|  | F2: Decision Making | .856 | .858 | .853 |
|  | F3: Goal Orientation | .737 | .758 | .696 |
| CFA sample  (*n* = 856) | F1: Self-Control | .866 | .868 | .862 |
|  | F2: Decision Making | .783 | .786 | .767 |
|  | F3: Goal Orientation | .761 | .775 | .719 |
| Female  (*n* = 1.356) | F1: Self-Control | .866 | .867 | .861 |
|  | F2: Decision Making | .773 | .778 | .759 |
|  | F3: Goal Orientation | .764 | .780 | .722 |
| Male  (*n* = 355) | F1: Self-Control | .855 | .857 | .855 |
|  | F2: Decision Making | .749 | .751 | .729 |
|  | F3: Goal Orientation | .762 | .772 | .714 |
| Age: 18-20 years  (*n* = 384) | F1: Self-Control | .843 | .845 | .845 |
|  | F2: Decision Making | .765 | .767 | .750 |
|  | F3: Goal Orientation | .766 | .791 | .728 |
| Age: 21+ years  (*n* = 1.327) | F1: Self-Control | .868 | .870 | .863 |
|  | F2: Decision Making | .770 | .775 | .755 |
|  | F3: Goal Orientation | .761 | .774 | .716 |
| Poland  (*n* = 276) | F1: Self-Control | .870 | .872 | .869 |
|  | F2: Decision Making | .719 | .734 | .740 |
|  | F3: Goal Orientation | .769 | .789 | .729 |
| Serbia  (*n* = 410) | F1: Self-Control | .871 | .875 | .871 |
|  | F2: Decision Making | .741 | .745 | .727 |
|  | F3: Goal Orientation | .705 | .722 | .663 |
| Slovakia  (*n* = 511) | F1: Self-Control | .849 | .852 | .848 |
|  | F2: Decision Making | .743 | .748 | .732 |
|  | F3: Goal Orientation | .787 | .807 | .758 |
| Czech Republic  (*n* = 514) | F1: Self-Control | .872 | .873 | .871 |
|  | F2: Decision Making | .799 | .803 | .786 |
|  | F3: Goal Orientation | .793 | .810 | .761 |
